# Optimization of operating parameters for biogas production using two-phase bench-scale anaerobic digestion of slaughterhouse wastewater: Focus on methanogenic step

**DOI:** 10.1186/s40643-022-00611-6

**Published:** 2022-12-09

**Authors:** Dejene Tsegaye, Seyoum Leta

**Affiliations:** Center for Environmental Science, College of Natural and Computational Sciences, Addis Ababa University, Addis Ababa, Ethiopia

**Keywords:** Methanogenesis phase, AD reactor stability and performance, Volatile solid reduction, Biogas production rate, Methane yield

## Abstract

The objective of the present study was an optimization of operating parameters and the performance of the methanogenesis reactor in phased anaerobic digestion (AD) of slaughterhouse wastewater at 37.5°C. Accordingly, the feedstock of the methanogenic reactor was effluent from the hydrolytic-acidogenic reactor operating at HRT of 3-days and OLR of 1789 mg/L. The methanogenesis phase was also investigated at different hydraulic retention time (HRT) values ranging from 12 to 3 days at 3-day intervals, and organic loading rates (OLR) of 149, 199, 298, and 596 mg of COD/L. The methanogenesis reactor effluent concentrations of TN, TP, PO_4_^− 3^, SO_4_^− 2^, and S_2_^− 2^ were ranging between 424–464, 83–117, 63–86, 130–197, and 0.98–1.02 mg/L, respectively. The removal efficiencies of TN and TP were vary from 10–17% to 17–21%, respectively. The average biogas production was 125 ± 16, 150 ± 10, 185 ± 4, and 154 ± 17 mL at HRT of 12, 9, 6, and 3 days, respectively. Methane quality (%) and yield (mg/L of COD) were 55–67% and 0.02–0.03, respectively. Furthermore, the average stability indicator parameter values of (total volatile fatty acid (TVFA) = 520 ± 19 mg/L, total alkalinity (TotA) = 1424 ± 10 mg/L, TVFA:TotA. Ratio = 0.36, salinity = 1172 mg/L, pH = 6.92) and performance indicator parameters removal efficiency (RE) for (chemical oxygen demand (COD) = 81%, volatile solid (VS) RE = 95%, biogas production = 185 ± 4 mL, methane yield = 0.03 per mg COD consumed) were achieved at HRT of 6 days and OLR of 298 mg of COD/L. Low removal efficiencies of TP and TN at all HRT/OLR were observed for the methanogenic reactor signifying further treatment system.

**Graphical Abstract:**

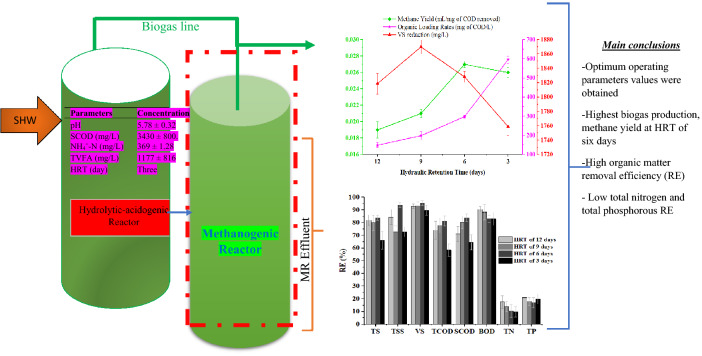

## Introduction

The slaughterhouse industry investment both for local service and export is increasing in Ethiopia, which is mainly associated with the livestock resources of the country, as it ranked first and 2nd in the horn of Africa region and the whole of Africa, respectively (Berhanu et al. 2019). In their nature, slaughterhouses are among industries characterized by water-consuming agro-processing industries. The wastewater generated from the slaughterhouse is mainly released from livestock receiving and washing (care), slaughtering operation, separation of the carcass from offal products, cleaning of stomach materials and intestine, sanitation, and other services like floor washing though the amount generated at each stage depends on the type of livestock slaughtered (Bustillo-Lecompte and Mehrvar 2015). Padilla-Gasca et al. (2011) reported the amount of wastewater generated per cattle is 700 L plus 25% of this for further processing of the edible meat. 18200000 m^3^ of wastewater is being generated from slaughterhouse industry sectors in Ethiopia. The wastewater mainly contains manure and urine, blood, stomach materials, and wash waters (Hernández et al. 2018). Slaughterhouse wastewater is high in suspended solids (SS) (3835–8000 mg/L), insoluble and soluble organic concentration that exhibits high COD (4000–11547 mg/L), and BOD (1200–4500 mg/L); and is categorized under strong wastewater (Worku and Leta 2017). Moreover, it also contains high phosphorous (30–202 mg/L) and nitrogen (95–1200 mg/L) (Bustillo-Lecompte et al. 2014; Aleksić et al. 2020; Mulu and Ayenew 2015; Kundu et al. 2013; Nweke et al. 2014).

Poorly managed slaughterhouse wastewater causes contamination of water, and soil (Abdullahi et al. 2015; Bello and Oyedemi 2009). In most developing countries including Ethiopia, management practices by many slaughterhouses are disposing to landfill or nearby water bodies which in turn poses major environmental challenges like bad odor, leachate management, eutrophication of water bodies, and greenhouse gas emissions. One such example is Organic export Abattoir private limited company found in Modjo town, 70 km away from Addis Ababa which is dedicated to processing and exporting mainly sheep and goat organic meat. About 800–1500 sheep and goats (each) per day are being slaughtered at this slaughterhouse for which a total of 400-L of water/sheep/goat is being used. An almost equivalent amount of wastewater is discharged into the nearby Modjo River without proper treatment increasing the pollution load on Koka Lake, the destination of the Modjo River. This is mainly due to the scarcity of technical and financial resources for wastewater treatment facilities and low regulations from concerning government bodies among others.

Capturing energy sources from slaughterhouse wastewater through biological conversion processes has received increasing attention in recent years. However, due to the high biodegradable, fat, and fibers contents treatment of slaughterhouse wastewater to the desired level is difficult mainly in single-phase anaerobic digesters which suffer from the accumulation of volatile fatty acids and ammonia inhibition that in turn decrease the biodegradation and biogas yield. In anaerobic biotechnology various configurations of reactors have been investigated and used to decrease the digestion time, required land space, and increase organic loading rate to maximize the biogas yield and removal efficiencies in wastewater. A two-phase AD system is at the forefront of the technology (Van et al. 2020; Dinopoulou 1988; Tanarat and Hanjai 2020).

Hence, optimization of two-phase digestion processes at each step is necessary due to the growth differences of the hydrolytic-acidogenic (HR) and methanogenesis (MR) reactors’ bacteria characteristics. Hydrolytic-acidogenic and methanogenic reactors’ separation in the anaerobic digestion system supports the growth of bacteria groups at optimum operating conditions. Two-phase AD (physically separated reactors) are suitable for effluents with high biodegradable organic matter (Tanarat and Hanjai 2020). The phase separation helps to optimize operating parameters for both reactors based on the requirements of the consortium of bacteria, hence better process control. Possible overloading of a methanogenic reactor can be detected at the hydrolytic-acidogenic phase and prevented by the supply of the acidified effluent from HR at optimal employment of methanogenic activities present in MR (Wilson 2009).

The HR serves as buffering reactor by reducing the easily floating grease and oil and partially degrading the organic matters in the agro-industrial wastewater (Ghorbanian 2014). This in turn increases the stability of the methanogenesis reactor by avoiding the accumulation of TVFA by a sudden increase of OLR as acetogens grow slower than acidogenic (Tanarat and Hanjai 2020). Furthermore, the second reactor is methanogen rich with an obligate anaerobic which is sensitive to the variation of operating conditions such as OLR and HRT. This therefore necessitates the optimization of the methanogenesis phase operating conditions (Dinopoulou 1988; Van et al. 2020).

To this end, phased AD has been given due attention to optimizing each reactor to attain the highest performance-transformation of organic matters in wastewater to biogas and pollutant removal efficiency (Van et al. 2020). To optimize the HR and MR reactors, it could be useful to engineer the operation of the HR towards acid formation which the methanogens prefer as a substrate and the MR to produce higher biogas and remove more pollutants (Eylem 2017; Ghorbanian 2014; Janesch et al. 2021). Furthermore, regardless of their current importance and upcoming potential, anaerobic wastewater treatment systems have not always cherished auspicious standing (McCarty 1964). Though the two-phase AD system process optimization was comprehensively studied, there is a research gap in the optimization of the reactors separately to maintain stability and better performances to attain enhanced pollutant removals and biogas production from anaerobic digestion of slaughterhouse wastewater. Therefore, the objective of this paper was to optimize the methanogenesis phase at different HRT and OLR to achieve better reactor stability and performance in terms of biogas production and pollutant removal efficiencies.

## Materials and methods

### Feedstock and inoculum for the experiment

Composite slaughterhouse wastewater was collected from the effluent discharge line of the Organic Export Abattoir found in Modjo town, Ethiopia. This slaughterhouse belongs to the conglomerate and is at the forefront of export-based activities in the Ethiopian meat export market. It has a capacity of slaughtering more than 800–1500 sheep and goats (each) per day and a total of 400-L of water utilized per sheep or goat. Almost an equal volume of wastewater was discharged into the nearby Modjo River, increasing the pollution load on Koka Lake, the destination of the Modjo River. 20-L acidified polyethylene plastic containers ‘jerricans’ were used to collect and transport the wastewater sample to the Laboratory of Center for Environmental Science, Addis Ababa University. The wastewater sample was stored at 4°C for the physicochemical analysis before feeding to the hydrolytic-acidogenic reactor. The hydrolytic–acidogenic reactor was optimized at six HRT (6, 5, 4, 3, 2, and 1 day (s)) and equivalent OLRs at a mesophilic temperature of 37.5°C for the key parameters (SCOD, TVFA, pH, and NH_4_^+^-N) and the optimum operating condition was obtained at HRT of 3 days as described in (Bedane et al. 2020). The effluent from the hydrolytic-acidogenic reactor previously optimized, i.e., HRT of 3 days was used as a feedstock for the present study, i.e., methanogenesis phase stability and performance indicating parameter optimization at bench-scale. The inoculum used for the methanogenesis phase in the present study was obtained from Saint George Brewery Industry up flow anaerobic sludge blanket (UASB) wastewater treatment plant effluent operating at 37°C

### Bench-scale experimental setup (reactors design)

**Fig. 1:**
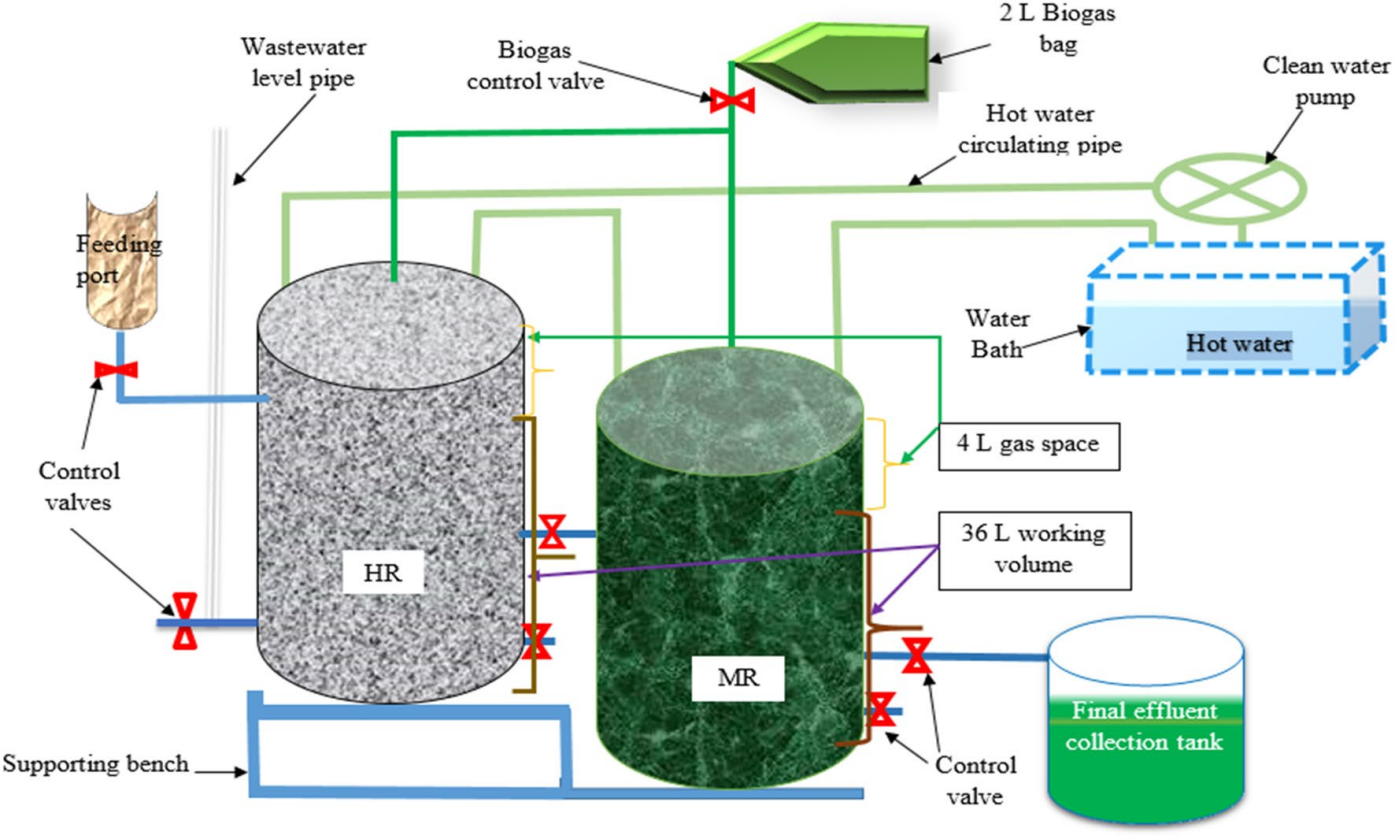
Schematic diagram of the bench-scale two-phase AD experimental setup

The optimization of the methanogenesis phase was carried out using a 40-L galvanized metal reactor (digester). The working volume and gas space of the reactors was 36-L and 4-L, respectively. A gasket maker was used to seal the reactors so that an anaerobic condition was created and tensioning bolts were used to strengthen the sealing. A thermostat water bath (Hangzhou West Tune Trading Co., Ltd, Zhejiang City, China) was used to maintain the reactor's temperature at 37.5°C. A Clean water pump (inGCO Inc., Zhejiang City, China) was used to pump while pipes composed of stainless steel inside the reactor and ¾ PPR pipe for the extension of the pipe outside the reactor were used to circulate the hot water from the thermostat water bath. The MR receive effluent from the hydrolytic-acidogenic reactor via ½ inch PPR pipe. A control valve was used to discharge the effluent from the hydrolytic-acidogenic to the methanogenesis reactors and another control valve extended on the connection point was used to take the effluent sample from HR. The reactors have also level regulation tubes and sludge discharging ports with control valves on HR and MR as indicated in Fig. 1.

### Operating conditions

During the two-phase anaerobic digestion of Organic Export Abattoir wastewater for biogas production, two bench-scale reactors with a total volume of 40-L (36 and 4-L, working and headspace volume, respectively) sequentially connected with a pipe were established as shown in Fig. 1 to optimize the methanogenesis phase reactor stability and performance indicator parameters. To create an anaerobic condition, both reactors were sealed with a gasket maker and bubbled with inert gas (nitrogen gas) before starting the experiment to dissolve the oxygen in the digester. The effluent from the previously optimized hydrolytic-acidogenic reactor, i.e., HR effluent at HRT of 3 days and OLR of 1789 mg of COD/L was used as feedstock/influent of the methanogenesis reactor. The operating conditions of the MR are presented in Table 1. To initiate the methanogenesis phase, the reactor was fed with a 1:1 ratio of acclimatized inoculum from St. George Brewery Industry UASB reactor effluent sludge with the effluent from the hydrolytic-acidogenic reactor. The system was acclimatized with the gradual addition of the effluent from the hydrolytic-acidogenic phase until the reactor working level (36-L) was achieved. Since then, the effluent of MR was collected in the final effluent collection tank and HR effluent of 3 days HRT with OLR described in Table 1 was fed to MR by opening the control valves based on the MR HRT/OLR. Optimum methanogenesis phase stability (TVFA, TotA, TVFA:TotA ratio, salinity, NH_4_^+^-N, and pH) and performance (pollutant removal efficiencies and total biogas/methane production) indicator parameters conditions were evaluated at OLR of 149, 198, 298, and 596 mg/L COD of hydrolytic-acidogenic reactor effluent. The 2-L glucose bag was connected to both MR and HR (not to lose biogas produced at HR if any). The average MR effluent values of all the parameters understudy were evaluated under steady-state conditions. The steady-state condition was assumed to be achieved when the concentration/values of the parameters under study were within 10% variation and twenty-two (22) consecutive readings were taken for each parameters (TVFA, TotA, salinity, NH_4_^+^-N, SCOD, TCOD, and pH) within 24 hour interval after realization of the steady-state condition. Samples were taken at the 3-day interval and analyzed for TN, TP, PO_4_^− 3^, H_2_S, SO_4_^− 2^, CH_4_%, CO_2_%, TS, TSS, and VS.

**Table 1:** Methanogenesis phase operating parameters of two-phase AD

HRT of MR (days)	Overall HRT (days)	Working volume (L)	Q (inflow) (V/HRT) MR (L)	OLR (mg COD/L)
12	15	36	2.4	149
9	12	36	3	199
6	9	36	4	298
3	6	36	6	596

### Analyses

Physicochemical characteristics of the wastewaters from the Organic Export Abattoir, HR and MR effluents were analyzed following standard methods (APHA 2017). TCOD, SCOD, TN, NH_4_^+^-N, TP, PO_4_^− 3^, S^− 2^, and SO_4_^− 2^ were analyzed following HACH instructions using a spectrophotometer (HACH DR/3900 HACH, Germany). Oxidation–reduction potential (ORP) and pH were analyzed using a pH meter (JENWAY, Manchester, UK). Resistivity, salinity, electrical conductivity (EC) and total dissolved solids (TDS) were analyzed by multi-meter (EUTECH Instruments, Madrid, Spain). TS and VS were analyzed according to Standard Methods for the Examination of Water and Wastewater (APHA 2017) using an oven at a temperature of 105°C and 550°C, respectively. TVFA and TotA were analyzed using titration according to (APHA 2017) standard method. Total biogas production was measured by sucking the biogas collected in 2-L glucose bag using a 100-mL airtight syringe. The biogas composition was measured using a gas analyzer (Geotechnical instrument gas analyzer, Leamington Spa, UK).

### Data analyses

The data registered on the laboratory logbook were entered into the MS excel spreadsheet 2013 version for further statistical analysis. Statistical analysis for mean, standard deviation and one-way analysis of variance (ANOVA) at 95% confidence interval was also performed using Minitab statistical software (Fegade et al. 2013). Origin 2022 software (Origin Lab Cooperation, Northampton, MA, USA) was employed to draw graphs.

## Results and discussion

The characteristics of slaughterhouse wastewater and HR effluent at HRT of 3 days used as the feedstock/influent of the methanogenesis phase are provided in Table 2. As shown in Table 2, the TCOD, SCOD, and BOD values (mean ± SD) of slaughterhouse wastewater were 5366 ± 827, 4842 ± 827 and 2487 ± 595 mg/L, respectively. Slaughterhouse wastewater content in terms of TCOD, and BOD reported earlier ranged between 4753 ± 1156 and 7080 ± 227 mg/L, and 2110 ± 602–43911 ± 389, respectively (Mulu & Ayenew 2015; Ren et al. 2014; Worku and Leta 2017). The EC, salinity, TDS, pH, ORP, and TVFA of slaughterhouse wastewater were varied between 1348 and 1964 ppm, 1210–1628 ppm, 1165–1684 ppm, 6.80–7.39, − 101 to − 63 mV, and 435–1197 mg/L, respectively. Padilla-Gasca E et al. (2011) reported that, the high EC, salinity, and TDS content is mainly due to the dissolved ion content NH_4_^+^-N, SO_4_^− 2^, and NO_3_^−^-N of slaughterhouse wastewater. The average (Mean ± SD) BOD, TCOD, SCOD, TVFA, NH_4_^+^-N, and pH values of the HR effluent at HRT of 3 days used as MR influent were 1175 ± 19 mg/L, 4945 ± 24 mg/L, 3430 ± 83 mg/L, 1177 ± 12 mg/L, 369 ± 11 mg/L, and 5.8 ± 0.04, respectively.

**Table 2:** Slaughterhouse wastewater and MR influent (HR effluent at HRT of 3 days)/feedstock characteristics

Parameter	Slaughterhouse wastewater concentration	HR effluent/MR influent concentration
pH	7.06 ± 0.30	5.78 ± 0.04
Salinity (ppm)	1209 ± 428	1650 ± 12
Electrical conductivity (µS/cm)	1346 ± 463	1810 ± 12
Resistivity (Ω)	458 ± 156	318 ± 22
TDS (ppm)	1171 ± 400	1576 ± 107
ORP (mV)	− 63 ± 18	− 82 ± 7
TVFA (mg/L)	817 ± 382	1177 ± 12
BOD (mg COD/L)	2488 ± 595	1175 ± 19
TCOD (mg/L)	5366 ± 827	4945 ± 24
SCOD (mg/L)	4842 ± 826	3430 ± 83
NH_4_^+^-N (mg/L)	338 ± 58	369 ± 11

### Effect of HRT and OLR on stability indicating parameters of methanogenic phase

The methanogenic reactor stability was evaluated based on parameters such as TVFA, TotA, TVFA/TotA ratio, NH_4_^+^-N, pH, and ORP. Table 3 and Fig. 3 indicate the mean ± SD and variation of the reactor stability parameters for the methanogenic reactor, respectively.

**Table 3:** Methanogenesis reactor effluent stability indicator parameters values at different HRT and OLR

Parameter	HRT (days)			
	12	9	6	3
OLR (mg COD/L)	149	199	298	596
TVFA (mg/L)	541 ± 18	526 ± 45	520 ± 19	604 ± 26
TotA (mg/L)	1174 ± 45	1424 ± 63	1534 ± 11	1537 ± 78
TVFA:TotA ratio	0.46	0.35	0.36	0.39
Salinity (mg/L)	1172 ± 17	1311 ± 16	1172 ± 17	1224 ± 15
NH_4_^+^-N (mg/L)	362 ± 10	372 ± 53	382 ± 53	400 ± 55
Effluent pH	6.91 ± 0.2	6.90 ± 0.2	6.92 ± 0.04	6.53 ± 0.1
ORP (mV)	− 82 ± 4	− 67 ± 3	− 80 ± 3	− 73 ± 6

As indicated in Table 3, the TVFA, TotA, TVFA/TotA ratio, pH, and ORP values of MR varies from 604 to 541 mg/L, 1537–1173 mg/L, 0.46–0.35, 6.92–6.53 and − 82 to (− 67) mV, respectively.

### TVFA

VFA are short-chain fatty acids that are the key end product of the hydrolysis reactor in phased AD serving as a precursor for the methanogenesis reactor as a carbon source for the methanogens. This indicates the production of TVFA is high in the hydrolysis phase and low/decreased in the methanogenesis phase as they are consumed by methanogens. This consumption of the TFVA in the methanogenesis phase is a good indicator of both enhanced biogas production and reactor stability (Michael et al. 2020). The accumulation of VFAs in MR in most cases reflects the imbalance among acid producers and consumers bacteria which in turn causes the drop in pH of the reactor (Rajagopal et al. 2013a, b; Rocamora et al. 2020). The mean TVFA for all methanogenic reactors at different OLR/HRT is presented in Table 3. As revealed in Table 3 and Fig. 3, the concentration of TVFA was decreased at all HRT and its concentration increased as the HRT decreases from 12 to 3 days. In addition, the variation of TVFA was going with the variation of the reactor pH (Figs. 3, 4). In stable MR the TVFA decreases as they are used as a carbon source for the growth of methanogens in the two-phase AD systems (Michael et al. 2020). Additionally, the finding of the present study is also in line with the result reported by (Worku and Leta 2017). Furthermore, the present study finding is also in agreement with the finding by Padilla-Gasca et al. (2011) which showed a maximum TVFA concentration of 448 mg CH_3_COOH/L without altering system stability in their study of anaerobic treatment of slaughterhouse wastewater. But the present study finding regarding TVFA concentration of methanogenic reactor is lower than the value reported by Berhe and Leta (2018) and Padilla-Gasca et al. (2011) ranging from 790 to 980 mg CH_3_CHOOH/L for methanogenesis reactor in their study of two-phase anaerobic co-digestion of tannery and dairy wastewater in different mixing ratios. The lower TVFA value of the present study may be due to the mono digestion of the feedstock (slaughterhouse wastewater effluent alone).

**Fig. 2:**
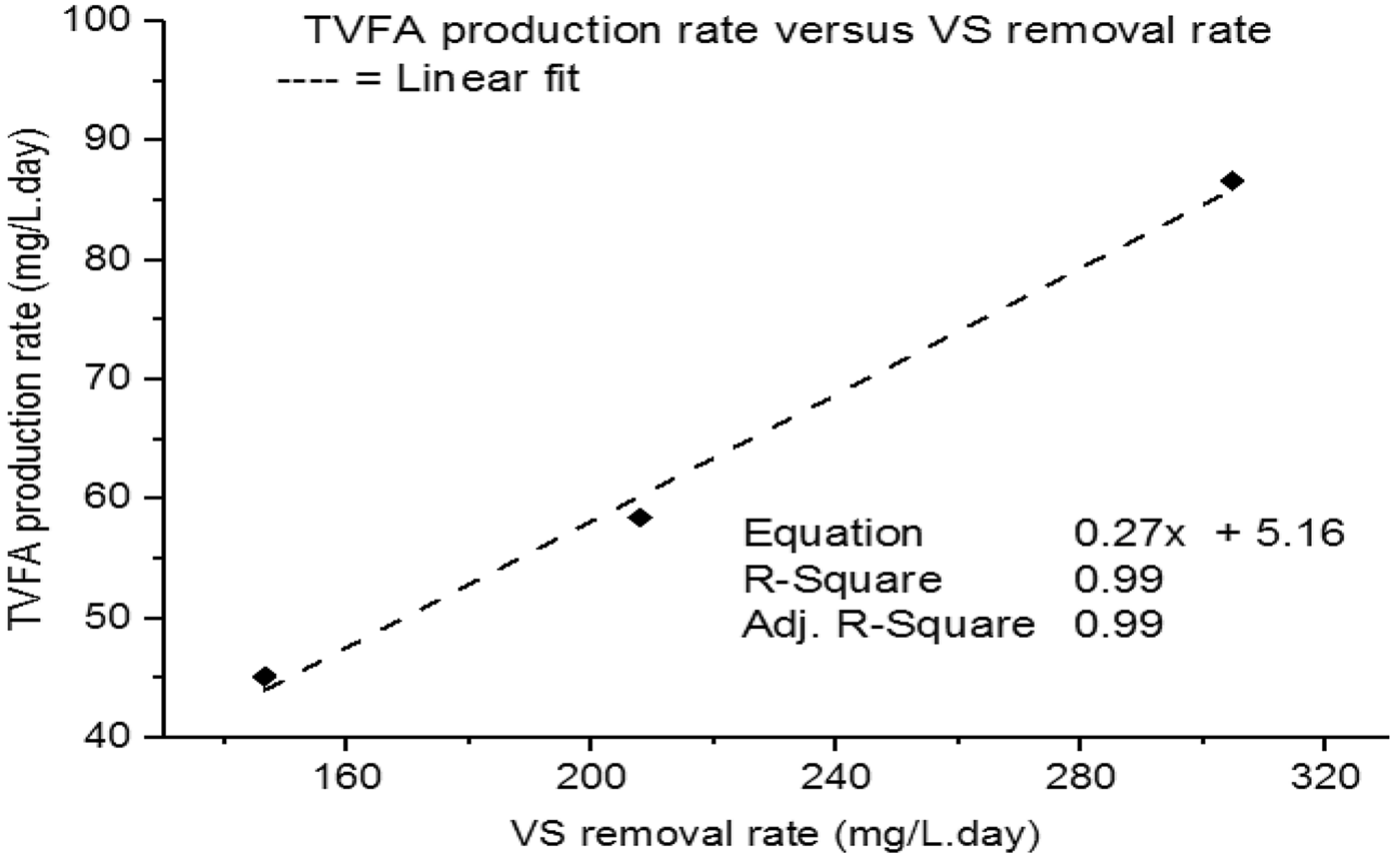
TVFA production rate verses VS removal rate

The TVFA production rate was determined as the TVFA concentration result and VS reduced, and decomposed. Accordingly, as shown in Fig. 2, the TVFA production rate was 5 mg/mg of VS removed. The TVFA concentration during the methanogenesis phase tended to be directly proportional to the organic matter (VS) removed (Padilla-Gasca et al. 2011). Similarly, the concentration of the TVFA concentration (production rate) is highly positively correlated (R^2^ = 0.99) to the VS reduction in the methanogenesis phase (Fig. 2). Moreover, the high linear correlation between TVFA production rate and VS removed of the MR shows that there is no high consumption of the intermediate (TVFA) or no TVFA accumulation, rather better reactor stability and performance during the two-phase AD process (Singharat et al. 2017).

### Total alkalinity

Total alkalinity plays an important role during the digestion process by buffering the acidity derived from the acidogenesis process in HR reactor during two-phase anaerobic treatment process to control the pH of MR. Cao et al. (2019) reported the TotA value within the acceptable range favors the production of biogas through buffering the reactor via maintaining the pH. The authors also stated that maintaining the alkalinity of the reactor within the favorable range is very important for optimal biogas production. In well-performing wastewater treating methanogenic reactor TotA and NH_4_^+^-N can be expected to increase as the result of the breakdown of protein into ammonia, which again combined with carbon dioxide to form ammonium bicarbonate (Sunirat Rattana, 2016).

1 $$NH_{3} \; + \;H_{2} O\; + \;CO_{2\;} \; \to \;NH_{4} \;\left( {HCO_{3} } \right)$$

**Fig. 3:**
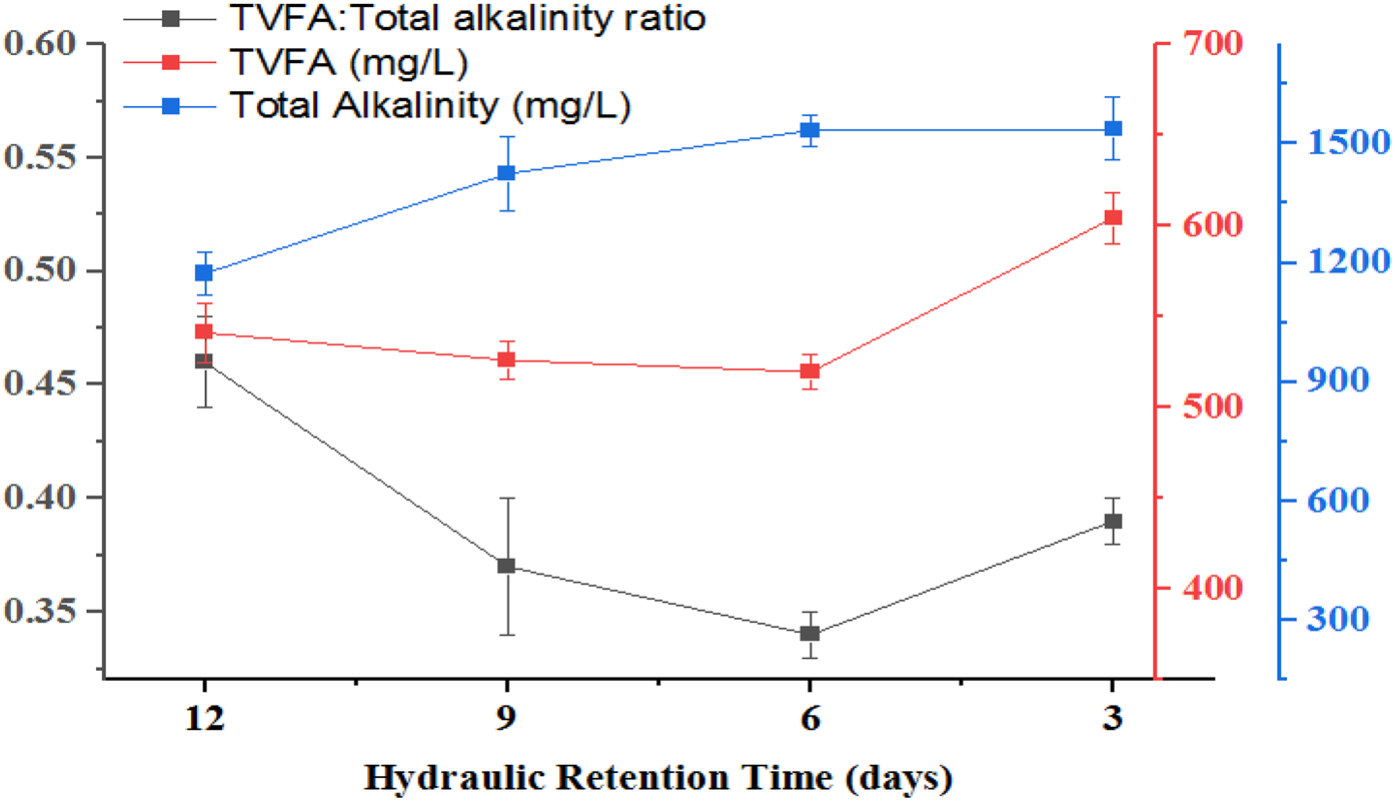
Average TVFA, TotA and TVFA/TotA ratio of MR at different HRT and OLR

The average influent and effluent TotA results of the methanogenic reactor/phase are shown in Table 3 and Fig. 3. The TotA of the reactor was gradually increased and stabilized with reaction time at all HRT indicating the reactor stability of the system (Berhe and Leta 2018; Padilla-Gasca et al. 2011). As HRT decreased from 12 to 3 days or OLR increased from 149 to 596 mg of COD/L, the average alkalinity value of the reactor was increased (Fig. 3), which is in line with the result reported earlier by Rocamora et al. (2020).

In well-performing or stable reactor, the TotA values ranges 1000–5000 mg CaCO_3_/L were reported (Berhe and Leta 2018). In the present study, the TotA values are in the range that favor reactor stability and enhances biogas production. This buffering capacity of the reactor recovers the hydrolytic-acidogenic reactor effluent pH of 5.8 and that of the start-up reaction period of MR to almost neutral which suggests the utilization of H^+^ by microbial in the reactor like hydrogenotrophic methanogens, chemolithotrophic sulfur oxidizing bacteria or/and oxidizing homoacetogens (Padilla-Gasca et al. 2011).

### TVFA/TotA ratio

Previous studies showed TVFA/TotA ratio is a parameter that is used to evaluate the anaerobic reactor stability at an early stage (Padilla-Gasca et al. 2011; Rincón et al. 2008). Accordingly, the present study also examined the TVFA/TotA ratio of the methanogenesis reactor at different HRT and the result showed that the ratio of the acidity to that of TotA varies between 0.46 and 0.34 (Table 3; Fig. 3). This can be due to the consumption of OM by microorganisms for the production of biogas (Padilla-Gasca et al. 2011). The TVFA/TotA ratio that falls within the 0.10–0.30 Barampouti et al. (2005); Padilla-Gasca et al. (2011), 0.30–0.40 Chen et al. (2015a, b); Fonoll et al. (2015); Rincón et al. (2009); Sindhu and Meera (2012) and above 0.40 Padilla-Gasca et al. (2011) indicates the avoidance of acidification, stability and instability of the process in the methanogenesis reactor, respectively. But the ratio values of the present study is in the stable methanogenesis reactor range (0.3–0.4) and showing high self-buffering capacity of MR except at HRT of 12 days (Table 3 and Fig. 3). The optimum TVFA/TotA ratio of stable and best-performing MR lies in the range of 0.3–0.4 (Chen et al. 2015a, b; Sindhu and Meera 2012; Fonoll et al. 2015; Rincón et al. 2009). Furthermore, elsewhere it was reported that the values of pH and ratio of TVA/TotA of 6.9 ± 0.04 and 0.35 ± 0.02, respectively, have high buffer capacity and less acidification risk hence leading to the high process stability of the methanogenesis reactor as the environmental condition in the AD process can control the system (Meesap et al. 2012; Grau et al. 1975).

### pH

**Fig. 4:**
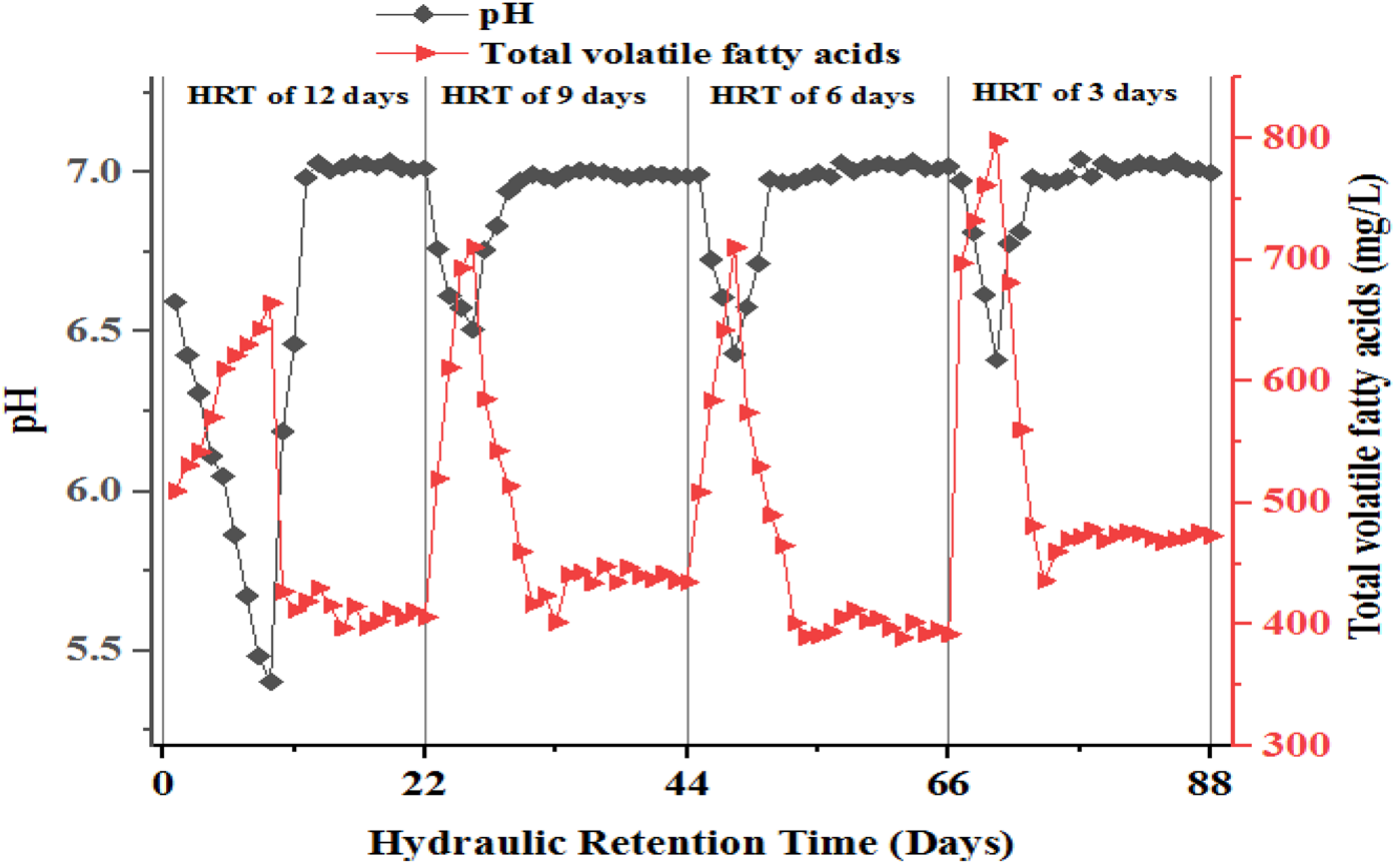
Variation of pH and TVFA in MR at different HRT and OLR

pH is an important stability indicator parameter of methanogenic reactors though it is associated with another parameter. The mean pH values of the methanogenesis reactor were 6.91, 6.90, 6.92, and 6.53 at HRTs of 12, 9, 6, and 3 days, respectively (Table 2). This indicates that, the mean pH value for the present study are near neutral and in the peak pH stability range of a methanogenic reactor, though they drop in the first few days of the experiment. Significant variations of pH were observed for HRTs of 12, 9, 6, and 3 days at *p* < 0.05 (Fegade et al. 2013). In addition, a similar trend was observed for methanogenic reactors operating at HRTs 12, 9, 6, and 3 days, i.e., a small decrement during the start-up period due to the accumulation of VFA and gradually rise due to better self-buffering capacity of the reactor as it receives partially treated effluent from HR and comes to steady state (Fig. 4). Methanogenesis/AD reactor operating at optimal condition pH range lies between 6.5 and 8.5 though the peak is near 7 (Rajakumar et al. 2012; Mao et al. 2017).

### Ammonium-nitrogen

The two most predominant forms of inorganic nitrogen are ammonium and free ammonia. In anaerobic wastewater treatment system ammonia is produced from protein, nitrogenous fat and nucleic acid degradation (Sung and Liu 2003) and is more toxic than ammonium as it passes through the cell membrane and into the cell causing potassium and proton imbalance of the methanogenic bacteria (Demirel et al. 2008) though acclimatized methanogens tolerate NH_4_^+^-N concentration of up to 2000 mg/L (Chen et al. 2016, 2008; Orhan and Burak 2013). At neutral pH ammonia is mainly found in the form of ammonium. In the present study, the NH_4_^+^-N concentration was investigated for the methanogenesis phase at different HRT/OLR and the mean value is provided in Table 3**.** As indicated in Table 3, the NH_4_^+^-N concentration ranges from 362 to 400 mg/L which is not in the range of inhibitory concentration level for the bacteria in the methanogenesis phase. Moreover, the present study finding also showed, a decrease in NH_4_^+^-N concentration as OLR decreases or HRT increases. Nakakubo et al. (2008) and Rocamora et al. (2020) reported that the concentration of NH_4_^+^-N in MR decreases with a decrease in OLR/increase of HRT. Methanogenesis reactor NH_4_^+^-N concentration of less than 200 mg/L is used as a nutrient source for the microorganism while a high level may cause a reduction in methanogens activity which in turn increase TVFA concentration and reduce methane production (Chen et al. 2008; Appels et al. 2008; Rajagopal et al. 2013a, b).

### Oxidation reduction potential (ORP)

ORP was also evaluated in the present study for the methanogenic phase and the mean ± SD for each OLR/HRT is presented in Table 3. As ORP is used to define the environment of biochemical reactions and the ORP obtained favors the methane reducing bacteria and inhibitory to sulfate-reducing bacteria which is in agreement by the finding reported by Duangmanee (2009). The negative ORP values indicate that reduced substances like methane and ammonia are produced from the degradation of the wastewater (Hailu et al. 2020). The negative value of ORP in the present study also shows the working condition, i.e., anaerobic type and an indicator of methane production possibility as also demonstrated by Vongvichiankul et al. (2017).

### Effect of HRT on reactor performance indicator

The methanogenesis phase performance evaluation was conducted for pollutant reduction/removal efficiencies (organic matter and nutrient), biogas production and methane yield.

### Organic matter removal efficiencies

#### Chemical oxygen demand and soluble chemical oxygen demand removal efficiencies

**Fig.5:**
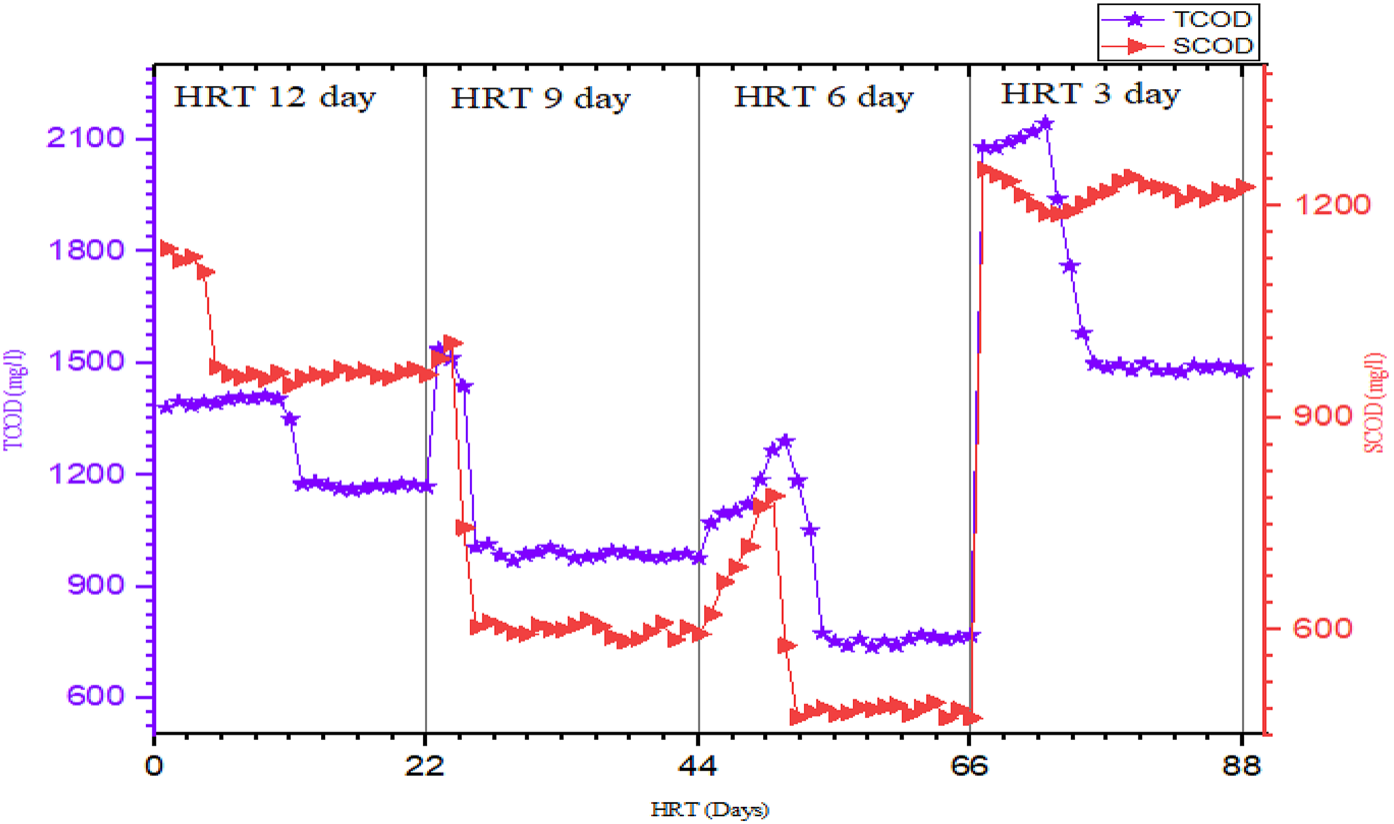
Trends/variation of COD and TCOD in MR at different OLR/HRT

**Fig. 6:**
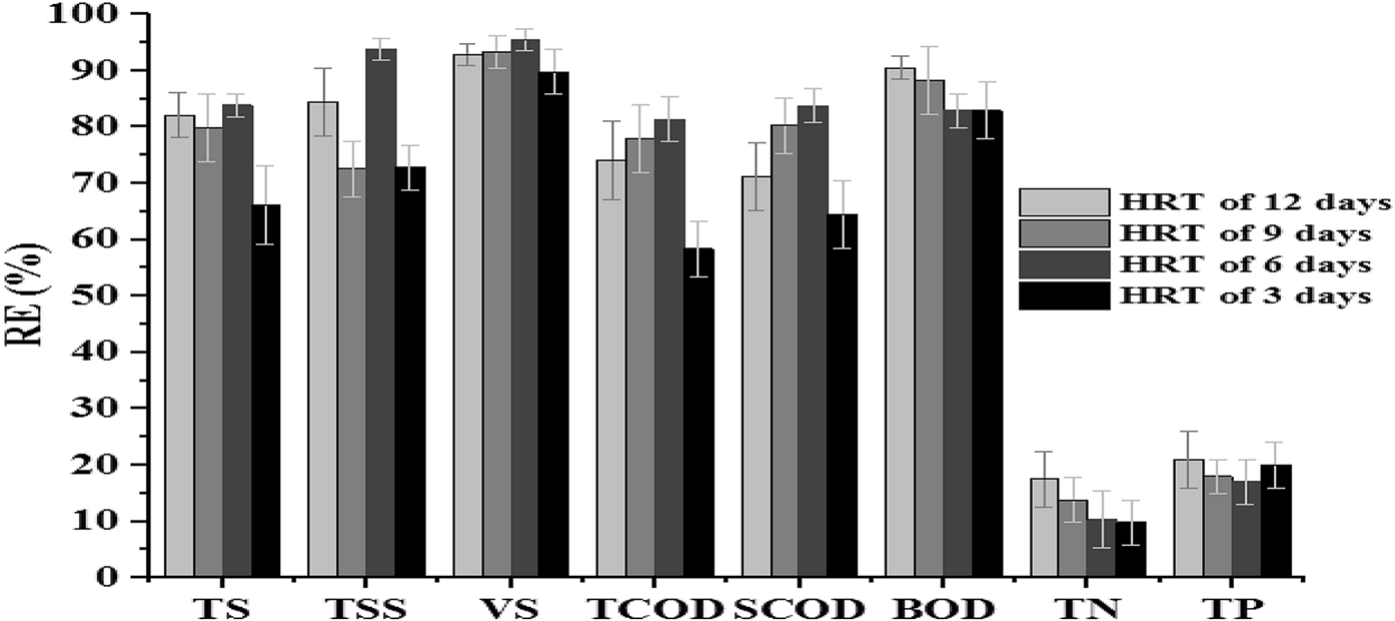
Organic matter, TN, and TP removal efficiency of methanogenesis reactor

TCOD and SCOD reduction and removal efficiencies were used to evaluate the methanogenesis phase reactor performance at different HRT and corresponding OLR. The TCOD and SCOD reduction at each HRT are indicated in Table 4. TCOD consumed were 3663 ± 13, 3852 ± 45, 4026 ± 36, and 2886 ± 38 mg/L at HRTs of 12, 9, 6, and 3 days, respectively. The result showed that TCOD removal efficiency increases as HRT decreases from twelve to six days and decreases as HRT decreases from six to three days and high TCOD removal efficiency (81%) was observed for the methanogenesis reactor operated at HRT of six days and OLR of 298 mg/L of COD (Table 4; Fig. 6). In addition, TCOD reduction was significantly varied among MRs’ operated at different HRT and OLR with a *p*-value of 0.00 (*p*-value <0.05) (Fegade et al. 2013). The variation of TCOD with reaction period (in days) for each HRT is indicated in Fig. 5. TCOD values were high during the start-up of the experiment, sharply decrease with the reaction period and comes to a steady state after twelve days of reaction time at HRT of 12, 6, and 3 days. Though higher values of TCOD were observed at HRT of 9 days, it drops sharply and comes to a steady state after a 13-day reaction period (Fig. 5**)**.

**Table 4:** Mean values of MR organic matter at all HRT and OLR

Parameter	HRT in days			
	12	9	6	3
Influent TCOD (mg COD/L)	4945 ± 24	4945 ± 24	4945 ± 24	4945 ± 24
Influent SCOD (mg COD/L)	3430 ± 83	3430 ± 83	3430 ± 83	3430 ± 83
Influent BOD (mg COD/L)	1175 ± 20	1175 ± 20	1175 ± 20	1175 ± 20
TS (mg/L)	232 ± 18	260 ± 46	209 ± 31	439 ± 102
TSS (mg/L)	88 ± 56	155 ± 102	35 ± 20	153 ± 112
VS (mg/L)	140 ± 45	131 ± 37	89 ± 23	200 ± 18
Effluent TCOD (mg/L)	1281 ± 12	1093 ± 29	919 ± 21	2059 ± 46
Effluent SCOD (mg/L)	991 ± 65	679 ± 16	555 ± 11	1219 ± 17
Effluent BOD (mg/L)	111 ± 45	137 ± 34	166 ± 51	200 ± 36
TCOD removed (mg/L)	3663	3852	4026	2886
VS removed (mg/L)	1759	1870	1828	1819
RE TS (%)	82	80	84	66
RE TSS (%)	84	73	94	73
RE VS (%)	93	93	95	90
RE TCOD (%)	74	78	81	66
RE SCOD (%)	71	80	84	64
RE BOD (%)	91	90	87	85

Another parameter used in the present study for the performance investigation of MR at different HRT was SCOD. The mean values of SCOD and trends/variation with reaction period for different HRT time are presented in Table 4 and Fig. 5, respectively. As HRT decreases from twelve to six days the SCOD reduction was improved but further decreases in HRT decline the removal efficiency of SCOD. As seen in Table 4, the highest and lowest SCOD removal were recorded for the MR operated at HRT of 6 and 3 days, respectively. From the trend graph of SCOD against the reaction period, the steady-state condition for SCOD was achieved early when compared to TCOD at each HRT under study. The highest and lowest BOD removal efficiency for the methanogenesis phase was achieved at HRT or OLR of 12 and 3 days or 149 and 596 mg of COD/L, respectively. In general, the finding of the present study shows MR operated at HRT of six days and OLR of 298 mg of COD/L showed high-performance in terms of all organic matter removal efficiency except for BOD.

The increase in TCOD and SCOD removal efficiency as HRT decreases from 12–6 days or OLR increases from 149 to 298 mg of COD/L may be attributed to the optimal microorganisms’ activity of the methanogenesis phase though longer HRT usually allows enough contact time for the microorganism with the partially treated wastewater in HR so that the decomposition of the organic matter by the system becomes efficient (Utami et al. 2016). Studies also indicate biomass drift-out and microorganism granulation are the drawbacks of the anaerobic reactors operating at short and long HRT, respectively (Demirer & Chen 2004; Utami et al. 2016; Worku and Leta 2017). Moreover, the later reported a similar effect of OLR/HRT on TCOD and SCOD removal efficiency during AD of slaughterhouse wastewater. To this end, the organic matter (TCOD, SCOD, BOD, and VS) were reduced as they were hydrolyzed and degraded to TVFAs by hydrolytic bacteria and acid-forming bacteria, respectively, in the hydrolytic-acidogenic reactor then converted to biogas by methanogens in the methanogenesis reactor/phase (Zhang et al. 2014; Demirer and Chen 2004).

#### Total solid and volatile solid removal efficiencies

The average reduction and removal efficiency of organic matter (TS and VS) are shown in Fig. 6 and Table 4, respectively. The result shows a significant variation of TS and VS removal efficiencies for HRTs of 12, 9, 6, and 3 days with F-values of 13 and 8, which is greater than the corresponding *p*-values of 0. 000 and 0.004, respectively (*p* < 0.05) (Fegade et al. 2013). The lowest (66%) and highest (84%) removal efficiencies of TS were observed at HRT of three and six days, respectively (Table 4). The VS removal efficiency of the MR was increased from 93 to 95% as HRT decreased from twelve to six days and a decrease in removal efficiency from 95 to 90% was observed for further decreasing of HRT. The effect of HRT or OLR on VS RE is comparable with the finding reported by Demirer and Chen (2004) during their study of the effect of HRT and OLR on bio-gasification. The higher VS removal efficiency than TS in the two-phase AD system at all HRT for methanogenesis reactor in the present study is mainly due to the high uptake of the organic fraction of total solids in the effluent of HR (Singharat et al. 2017; Zhang et al. 2014). Furthermore, VS removal efficiency was negatively correlated with OLR (Fig. 9).

#### Total nitrogen, total phosphorous and sulfate level of methanogenesis reactor effluent

The average TN of methanogenesis reactor effluent concentration are presented in Table 5. Accordingly, the average concentration of TN was 464 ± 32, 443 ± 35, 461 ± 41, and 464 ± 32 at HRTs of 12, 9, 6, and 3 days, respectively. The removal efficiencies of TN were 10, 10, 17, and 14% at HRTs of 12, 9, 6, and 3 days, respectively (Table 5; Fig. 6).

**Table 5:** Average TN, TP, PO_4_^− 3^, SO_4_^− 2^ and S^− 2^ concentration of methanogenesis reactor effluent at different HRT

Parameter	HRT in days			
	12	9	6	3
TN (mg/L)	464 ± 32	443 ± 35	461 ± 41	464 ± 34
TP (mg/L)	100 ± 17	103 ± 7	105 ± 7	101 ± 8
PO_4_^−3^ (mg/L)	63 ± 6	70 ± 5	75 ± 8	86 ± 10
SO_4_^−2^ (mg/L)	130 ± 30	146 ± 9	166 ± 4	197 ± 14
S^−2^ (mg/L)	0.98 ± 0.04	1.00 ± 0.01	1.01 ± 0. 01	1.02 ± 0.01

The average methanogenesis reactor effluent concentration of TP at HRTs of 12, 9, 6, and 3 days were 100 ± 17, 103 ± 7, 105 ± 7, and 101 ± 8 mg/L, respectively (Table 5). The highest and lowest TP concentration (mean ± SD) of the MR effluent were 105 ± 7 and 100 ± 8 at HRT of six and twelve days, respectively. The maximum and minimum removal efficiencies of TP were 21% and 17% at HRT of twelve and six days, respectively. The removal efficiencies for TP were 22, 18, 17, and 20% at HRTs of 12, 9, 6, and 3, respectively (Fig. 6). The decrease in MR effluent TP concentration is mainly due to the synthesis of biomass in the course of the AD process. In Marcin (2022), it was also stated that the decrease in TP concentration in the AD system was attributed to microbial activity and cell formation. The average PO_4_^− 3^ concentrations at HRTs of 12, 9, 6, and 3 days were 63 ± 6, 70 ± 6, 75 ± 8, and 86 ± 10 mg/L, respectively (Table 5). The high and low PO_4_^− 3^ concentrations of 86 ± 10 and 63 ± 6 mg/L were recorded at HRT of 3 and 12 days, respectively. Likewise, the variation of the PO_4_^− 3^ level of MR effluent at different HRT and OLR is significant at *p* < 0.05 (Fegade et al. 2013).

The average SO_4_^− 2^ and S^− 2^ concentrations of MR effluent are presented in Table 5. The average of SO_4_^− 2^, and S^− 2^ concentration varied from 130–197 to 0.98–1.02, respectively. The decrease in the concentration of SO_4_^− 2^ at the methanogenic phase is mainly due to the anaerobic microbial process (sulfate reduction). This sulfate reduction was mainly attributed to the hydrolytic-acidogenic reactor which acts as the sulfidogenic-acidogenic reactor in phase-separated AD (Janesch et al. 2021; Mburu et al. 2012). Furthermore, a comparable conclusion was drawn with the finding of the present study for the SO_4_^− 2^ and S^− 2^ effluent concentrations of AD by different scholars in treating slaughterhouse and other agro-industrial wastewater using an anaerobic reactor via biogas production (Alemu et al. 2019; Toledo et al. 2016) due to the low synthesis of bacteria or sulfate reduction process in methanogenesis phase in particular and in AD system in general (Sindhu and Meera 2012) recommending further biological treatment system requirement of post-AD.

#### Biogas production, methane content and yield

##### Biogas production

The trends of biogas production at the entire HRT of the methanogenic phase are shown in Fig. 8. At all HRT the biogas production was low in the start-up of the experiment, gradually increase and comes to a steady-state after the 15th day. The low biogas production at start-up periods was mainly due to the lag phase of microbial growth as the biogas production in the batch condition is directly equal to the specific growth of the methanogenic bacteria in the reactor. The gradual increase in biogas production for all HRT may attribute to the exponential growth of the methanogens. The average biogas production of the methanogenesis phase at different HRTs is presented in Table 2. The average biogas production was 125 ± 16, 150 ± 10, 185 ± 4, and 154 ± 17 mL at HRTs of 12, 9, 6, and 3 days, respectively. Biogas production increase from 125 to 185 mL as HRT decrease from twelve to six days but a further decrease of HRT or increase of OLR decreases the biogas production (Fig. 8). Significant variation of biogas production with HRT (*p*-value = 0.01) was observed for the methanogenesis phase at a 95% confidence interval (Fegade et al. 2013). The lower biogas production at the highest HRT/lowest OLR was mainly attributed to high consumption of the organic matter by methanogenic microorganisms for growth which resulted in the insufficient organic matter to be converted to biogas via reducing the biogas production. The lowest biogas of 125 ± 16 mL produced at HRT of three days was attributed to the methanogens activity due to the washout/overload during the discharge of the reactor effluent that causes process instability and reduction in biogas production (Wang et al. 2014; Vongvichiankul et al. 2017). The highest biogas production of 185 ± 4 mL at HRT of 6 days/OLR of 298 mg COD/L was mainly due to the maximum substrate utilization by the methanogen and the pH of the methanogenesis phase. The methanogens utilize maximum substrate at nearly neutral pH, which in turn favors high biogas production (Kavitha and Murugesan 2007; Rocamora et al. 2020). The biogas produced is comparable with the result reported by Demirer and Chen (2004) and Sindhu and Meera (2012) in the treatment and biogas production from the same feedstock. Moreover, increasing OLR can reduce the contact period of methanogenic bacteria consortia and feedstock (Hailu et al. 2020). Increasing the OLR/decreasing HRT up to a certain level increases the biogas production but further increase can decreases/do not affect biogas production (Hailu, Asfaw, and Tegaye 2020; Demirer and Chen 2004; Worku and Leta 2017; Berhe and Leta 2018; Kavitha and Murugesan 2007).

**Fig. 7:**
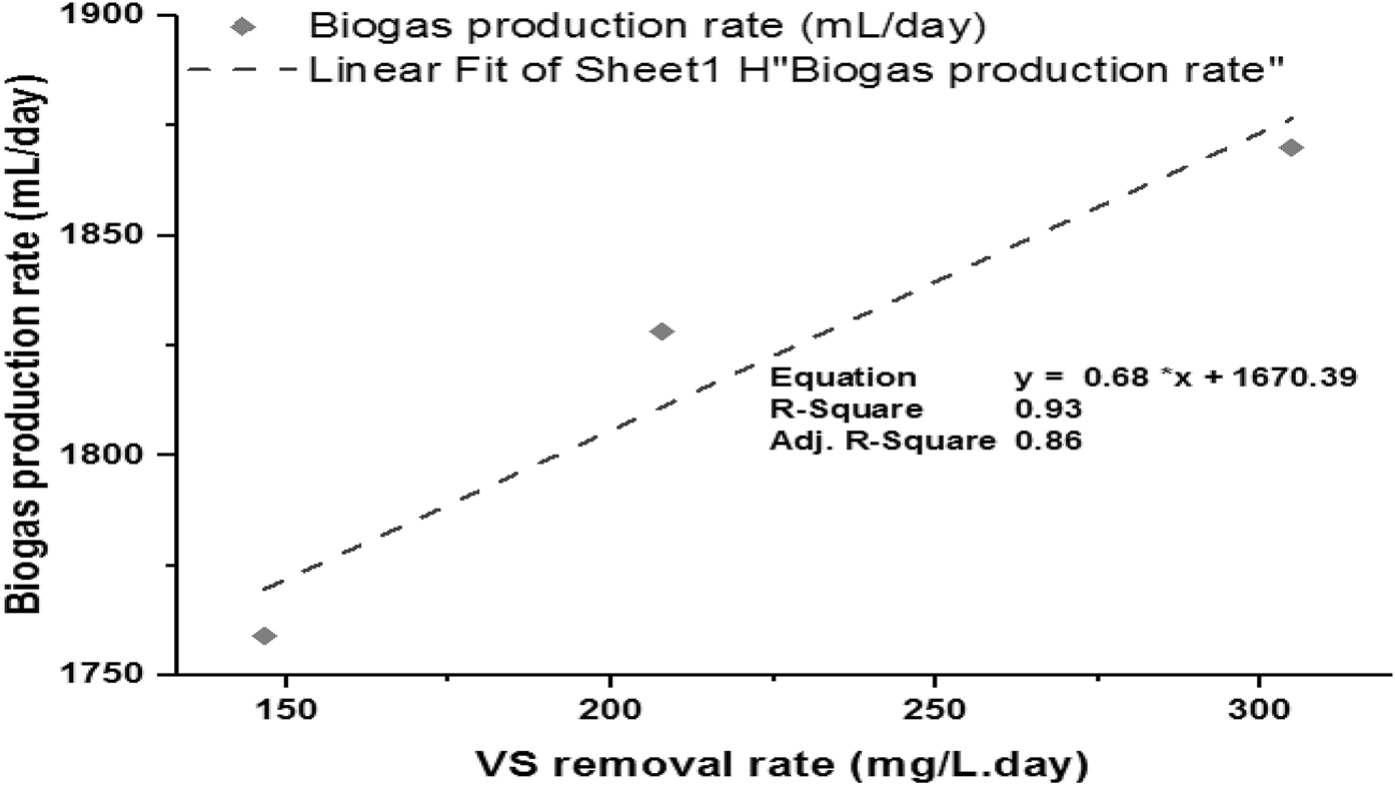
Biogas production rate

The biogas production rate was computed with VS reduced in the process. The biogas production was positively highly correlated (*R*^2^ = 0.93) with the VS removal showing that the process gained not only biogas production but also organic matter removal (Fig. 7). This shows the VS were transformed to TVFA and then converted to biogas in the methanogenesis reactor (Lee et al. 2015; Singharat et al. 2017).

##### Methane and carbon dioxide composition of biogas

**Fig. 8:**
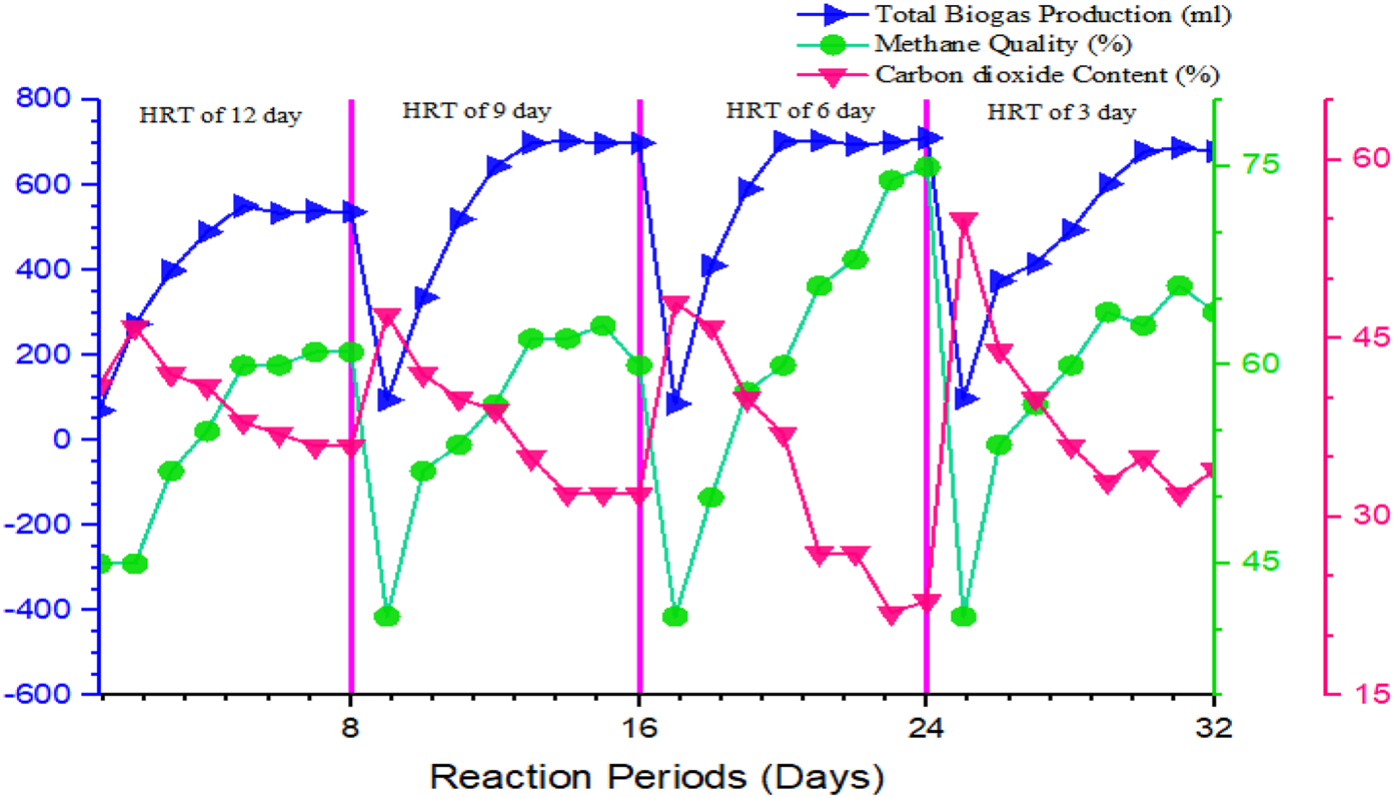
Variations of biogas production, methane and carbon dioxide percentage of methanogenesis reactor at different HRT

The methane content of biogas produced at all HRTs of the methanogenesis phase is illustrated in Fig. 8. As indicated in Fig. 8**,** low methane content/percentage was observed at the beginning of the reaction period and gradually increase and come to a steady-state condition at all HRT. The MR operated at HRT for six days showed good performance of 67% and 123 mL/day methane content and methane production rate, respectively. But MR operated at HRT of three and twelve days showed low performance (55%–70 mL/day) in terms of the methane content of the biogas produced and methane production rate, respectively. The decrease of HRT from twelve to six days increases the methane production rate from 70 to 123 mL/day. ANOVA test for the variation of methane content at HRTs of 12, 9, and 6 days was significant at (*p* < 0.05) (Fegade et al. 2013).

As shown in Fig. 8, the CO_2_ content in percent of biogas produced at the methanogenic phase was high at the start-up of the experiment and gradually decrease with time. The average CO_2_ content of biogas in percent (%) was 54 ± 4, 37 ± 7, 30 ± 12, and 36 ± 9 at HRTs of 12, 9, 6, and 3 days, respectively. The result shows that the CO_2_ content (%) of biogas produced decrease as HRT decrease or increase as OLR increase. Sindhu and Meera (2012) and Worku and Leta (2017) reported similar trends of biogas composition in their study of AD of slaughterhouse wastewater at different HRT and OLRs. Methane content of 43–63% was reported by Yilmaz (2007) in a phased AD system which is comparable to the finding of this study. Furthermore, Demirer & Chen (2004); Michael et al. (2020); Ortner et al. (2015) also reported equivalent methane content of 66–70% from AD of slaughterhouse wastewater. The substantial methane and lower carbon dioxide content of the present study at HRT of 6 days may be attributed to the operating condition, OLR/HRT and feedstock type in relation to the earlier findings reported. The methane and carbon dioxide content of biogas obtained from the AD of organic-rich feedstock varies from 50–75% to 25–45%, respectively (Michael et al. 2020). The same scholars also stated that the biogas composition of the biogas depends on the feedstock used for the anaerobic digestion and the methanogenesis bacteria consortia activity in the process which is the main reason for the lower result methane content and higher carbon dioxide content of biogas produced.

##### Methane yield

**Fig. 9:**
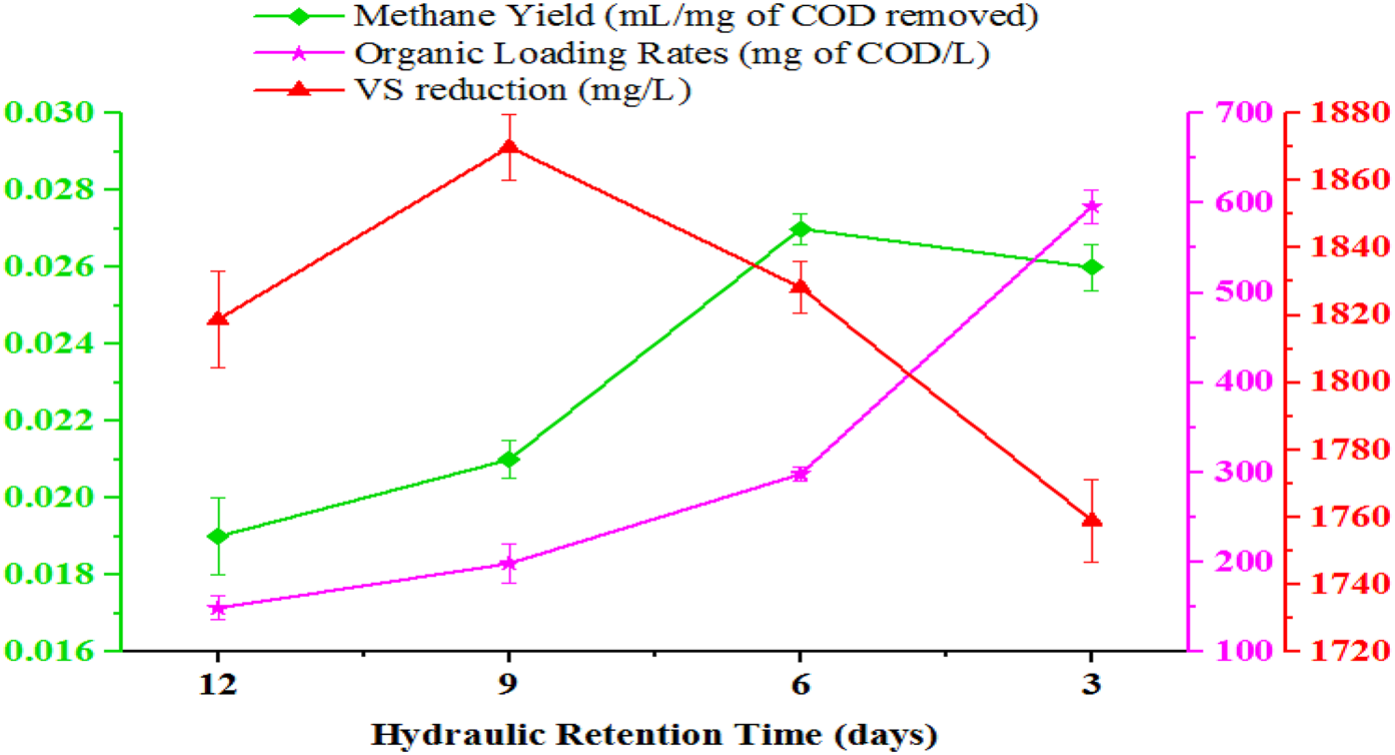
Variations of biogas production, methane and carbon dioxide percentage of methanogenesis reactor at different HRT

The average methane yield at all HRT of the methanogenesis phase is presented in Fig. 9. As shown in Fig. 9, methane yield was 0.019, 0.021, 0.027, and 0.026 mL per mg of COD removed at HRTs of 12, 9, 6, and 3 days, respectively.

Furthermore, the decrease in HRT from twelve to six days increases the methane production rate from 0.019 to 0.027 mL per mg COD removed (Fig. 9). The variation of methane content at different HRT was significant at (*p* < 0.05) (Fegade et al. 2013). The highest methane yield was recorded for a methanogenic reactor operated at HRT for six days and a corresponding OLR of 298 mg COD/L (Fig. 9). The increase in methane yield as HRT decreases or OLR increases is attributed to the maximal microorganism consortia activity of the methanogenesis phase and sufficient contact time with substrate/feedstock (Worku and Leta 2017; Sindhu and Meera 2012; Ahmad 2013; Demirer and Chen 2004).

The minimum and maximum HRT (OLR) the methanogenesis phase accommodated were 3 days (596 mg/L) and 12 days (149 mg/L), respectively. In general, the two-phase anaerobic digestion of slaughterhouse wastewater, MR, operated at an HRT of six days and an OLR of 298 mg of COD/L, showed high performance in terms of pollutant removal efficiency (except for BOD), biogas production, and methane yield. The system might be further improved by natural nanoparticle employment, effluent recirculation, and co-digestion.

## Conclusion

Methanogenesis phase stability and performance indicator parameters were investigated at different HRT and OLRTs at a constant temperature of 37.5°C. Accordingly, pH of nearly neutral (6.92) that favors the methanogens, TVFA:TotA ratio (0.36) which is in the optimum range, highest alkalinity that maintains the buffering capacity of the reactor and non-inhabiting concentration of NH_4_^+^-N (382 mg/L) obtained at HRT of six days and OLR of 298 mg COD/L. The TCOD and SCOD removal efficiency increases as HRT decreases from 12 to 6 days but a further decrease in HRT decreases the removal efficiency of both. The highest and lowest BOD removal efficiency was achieved at HRTs or OLR of 12 and 3 days or 149 and 596 mg of COD/L, respectively. The biogas production increase from 125 to 185 mL as HRT decrease from twelve to six days but a further decrease of HRT or increase of OLR decreases the biogas production. The highest biogas production of 185 mL was obtained at HRT of six days/OLR of 298 mg COD/L. Moreover, the methanogenesis reactor operated at HRT for six days showed good performance in terms of the methane content of the biogas produced (67%) and methane production rate (123 mL/day). The average CO_2_ content of biogas was 54 ± 4, 37 ± 7, 30 ± 12, and 36 ± 9 at HRTs of 12, 9, 6, and 3 days, respectively. The low removal efficiencies of TN (10–17%) and TP (17–21%) achieved signify post-AD treatment options.

## Data Availability

All the dataset and materials used for this manuscript are included in this document.

## Abbreviations

APHA: American Public Health Association
BOD: Biological oxygen demand
COD: Chemical oxygen demand
EC: Electrical conductivity
EPA: Environmental Protection Authority
HRT: Hydraulic retention time
NH_4_-N: Nitrogen ammonium
OLR: Organic loading rate
ORP: Oxidation reduction potential
SCOD: Soluble chemical oxygen demand
SHWW: Slaughterhouse wastewater
TCOD: Total chemical oxygen demand
TDS: Total dissolved solid
TN: Total nitrogen
TSS: Total suspended solids
TotA: Total alkalinity
TVFA: Total volatile fatty acids
VFA: Volatile fatty acids
